# Host-Symbiont Cospeciation of Termite-Gut Cellulolytic Protists of the Genera *Teranympha* and *Eucomonympha* and their *Treponema* Endosymbionts

**DOI:** 10.1264/jsme2.ME17096

**Published:** 2018-03-29

**Authors:** Satoko Noda, Daichi Shimizu, Masahiro Yuki, Osamu Kitade, Moriya Ohkuma

**Affiliations:** 1 Graduate School of Life and Environmental Sciences, University of Yamanashi Yamanashi 400–8510 Japan; 2 Biomass Research Platform Team, RIKEN Biomass Engineering Program Cooperation Division, RIKEN Center for Sustainable Resource Science Ibaraki 305–0074 Japan; 3 College of Science, Ibaraki University Mito, Ibaraki 310–8512 Japan; 4 Japan Collection of Microorganisms, RIKEN BioResource Center Ibaraki 305–0074 Japan

**Keywords:** cospeciation, Teranymphidae protist, endosymbiotic bacteria, *Treponema*, termite

## Abstract

Cellulolytic flagellated protists inhabit the hindgut of termites. They are unique and essential to termites and related wood-feeding cockroaches, enabling host feeding on cellulosic matter. Protists of two genera in the family Teranymphidae (phylum Parabasalia), *Eucomonympha* and *Teranympha*, are phylogenetically closely related and harbor intracellular endosymbiotic bacteria from the genus *Treponema*. In order to obtain a clearer understanding of the evolutionary history of this triplex symbiotic relationship, the molecular phylogenies of the three symbiotic partners, the Teranymphidae protists, their *Treponema* endosymbionts, and their host termites, were inferred and compared. Strong congruence was observed in the tree topologies of all interacting partners, implying their cospeciating relationships. In contrast, the coevolutionary relationship between the *Eucomonympha* protists and their endosymbionts was more complex, and evidence of incongruence against cospeciating relationships suggested frequent host switches of the endosymbionts, possibly because multiple *Eucomonympha* species are present in the same gut community. Similarities in the 16S rRNA and *gyrB* gene sequences of the endosymbionts were higher among *Teranympha* spp. (>99.25% and >97.2%, respectively), whereas those between *Teranympha* and *Eucomonympha* were lower (<97.1% and <91.9%, respectively). In addition, the endosymbionts of *Teranympha* spp. formed a phylogenetic clade distinct from those of *Eucomonympha* spp. Therefore, the endosymbiont species of *Teranympha* spp., designated here as “*Candidatus* Treponema teratonymphae”, needs to be classified as a species distinct from the endosymbiont species of *Eucomonympha* spp.

Numerous insects have established intimate mutualistic relationships with microorganisms, which play key roles in host adaptation to various environments (16, 40). This symbiotic relationship with microorganisms has a profound impact on the adaptation of termites to their xylophagous, wood-feeding lifestyle, and also contributes to the expansion of the termite niche in terrestrial ecosystems. Cellulolytic flagellated protists in the termite hindgut are unique to termites and related wood-feeding cockroaches, and are essential for host feeding on cellulosic matter (2, 31).

The family Rhinotermitidae includes the global pest genera of termites, such as *Coptotermes*, *Reticulitermes*, and *Heterotermes* (20). In this termite family, cellulolytic protists from the genus *Pseudotrichonympha* (phylum Parabasalia) are widely distributed as gut symbionts (17). The cells of *Pseudotrichonympha* protists harbor endosymbiotic bacteria of a unique phylogenetic lineage within the order Bacteroidales (22). The members of this triplex symbiotic system appear to have cospeciated during their evolution (25), possibly because of the crucial roles played by symbionts in termite nutrition (13, 33). Various examples of species-specific symbiotic relationships between protists and bacteria have been identified in the termite gut, and include ectosymbioses on the protist cell surface (29); many ectosymbionts are Bacteroidales and Spirochaetes members (14, 21, 23). As expected for endosymbioses, most, if not all, have cospeciated (15, 25, 39, 41), with some exceptions (10); ectosymbiotic relationships appear to be less strictly cospeciated than endosymbiosis (26).

The composition of the gut protist fauna is specific to the host species, and is suggested to reflect the host’s phylogeny (18). However, in the family Rhinotermitidae, termites from the genus *Reticulitermes* typically lack *Pseudotrichonympha* protists. The gut protist composition in this genus is unique within the Rhinotermitidae, and is similar to that of the phylogenetically distant genus *Hodotermopsis* from the family Archotermopsidae, an early branching group of termites (1). These two termite genera are often distributed sympatrically, and the Japanese species *Reticulitermes speratus* or *R. amamianus*, and *Hodotermopsis sjostedti* frequently coexist in the same fallen log (18). A previous study reported that the protist fauna of a *Hodotermopsis* termite may have been laterally transferred to *Reticulitermes* termites (18). *Hodotermopsis* and *Reticulitermes* termites are both characterized by the rich species diversity of gut protists, and commonly harbor members of the family Teranymphidae (phylum Parabasalia) as large-size cellulolytic protists. Two genera of Teranymphidae, *Eucomonympha* and *Teranympha* (5), are phylogenetically closely related and inhabit *H. sjostedti* and *Reticulitermes* termites, respectively (27).

*Eucomonympha* and *Teranympha* protists both harbor bacterial endosymbionts (32). In *H. sjostedti*, a rod-shaped endosymbiont of *Eucomonympha* sp. was shown to be a spirochete species from the genus *Treponema* and was designated as “*Candidatus* Treponema intracellularis” (32). Recent biochemical and single-cell genome analyses revealed that this endosymbiotic species plays crucial roles in termite nutrition, *e.g.*, reductive acetogenesis from H_2_ plus CO_2_ and nitrogen fixation (32). The former provides acetate as a major energy source for termites, while the latter provides nitrogen sources, which are poor in the termite diet (3). These dual functions of the endosymbiont as well as the cellulolytic ability of the protist may enhance the adaptability of the host termite to xylophagy, and are also responsible for the stability of the protist in the gut. In the gut of *R. speratus*, the endosymbiont species of *Teranympha mirabilis* was shown to be closely related to the endosymbiont of *Eucomonympha* sp. (32). Nevertheless, the distribution and phylogenetic relationships of the endosymbiotic *Treponema* among Teranymphidae protists have not yet been investigated.

In the present study, we identified the endosymbionts of Teranymphidae protists in *Reticulitermes* and *H. sjostedti* termites. The molecular phylogenies of the three symbiotic partners, Teranymphidae protists, their host termites, and their *Treponema* endosymbionts, were inferred and compared in order to obtain a clearer understanding of the evolutionary history of this triplex symbiotic relationship.

## Materials and Methods

### Data collection

Protist samples investigated in the present study and their host termites are listed in Table 1. All termite samples were collected in Japan (Fig. S1) and maintained in plastic boxes before use. Living termites were used as the material in the phylogenetic analyses of insects, protists, and endosymbiotic bacteria.

Termite DNA was extracted from the head and legs, and used as a template for PCR, as described previously (25). Mitochondrial COI, COII, and 16S rRNA genes were amplified, and used directly for DNA sequencing, as described previously (25). Teranymphidae protists were consistently found in the hindgut flora of the respective termites and were easily recognizable based on their morphological characteristics (6). The protist cells in the hindgut suspension of each termite were isolated manually and washed extensively under a microscope equipped with a micromanipulator (Cell Tram; Eppendorf, Hamburg, Germany), as described elsewhere (25). Each cell showing a typical morphology was isolated and was used as a template for isothermal whole-genome amplification (WGA), as previously described (27); amplified genomic DNA was used as a template for PCR. Regarding the expressed small subunit ribosomal RNA (SSU rRNA), 20 cells were directly used as a template for first-strand cDNA synthesis with the primer PZR1 (25, 27) and the resulting cDNA was used for PCR amplification.

The protist gene encoding SSU rRNA, and bacterial 16S rRNA and *gyrB* genes were amplified using the WGA sample prepared from the same isolated cell as a template. The three genes were amplified by PCR, using the high fidelity DNA polymerase KOD Plus Neo (Toyobo, Osaka, Japan), with the previously described primers (25). The amplified products were separated by agarose gel electrophoresis, purified, and then cloned using the Zero Blunt pCR4-TOPO PCR cloning kit for sequencing (Invitrogen, Carlsbad, CA, USA) and Competent Quick DH5α (Toyobo). Clones containing inserts of the expected size were picked and partially sequenced, and the complete DNA sequence of each representative clone was obtained. In each sample, 3–9 clones were analyzed for the *Teranympha* SSU rRNA gene, while 1–4 clones were analyzed for the *Eucomonympha* SSU rRNA gene. Most of the clone sequences of *Eucomonympha* spp. were almost identical to either one of the three phylotype sequences identified in the whole gut community of *H. sjostedti* (unpublished data).

Eight clones were typically analyzed for the bacterial 16S rRNA and *gyrB* genes in each WGA sample; the partial DNA sequences of clones were sorted into phylotypes at >98.5% (16S rRNA gene) and >99.4% (*gyrB* gene) sequence similarities. The 16S rRNA gene sequences that were distantly related to those of endosymbiotic *Treponema* were excluded from further analyses. In the case of *gyrB*, the phylotype sequences sharing high sequence similarities with the *Treponema* sequences were phylogenetically analyzed (Fig. S2), and only the sequences identified as the endosymbiotic *Treponema* species were concatenated for the phylogenetic analysis. The sequences obtained in this study have been deposited in the DNA Databank of Japan and the accession numbers are shown in Tables S1 and S2.

### Phylogenetic analyses

The DNA sequences obtained in the present study and publicly available sequences were aligned using ClustalW2 (19) and alignments were refined manually. Ambiguously aligned positions were omitted from the subsequent phylogenetic inference analysis. An appropriate model of sequence evolution was selected using the program Jmodeltest 2.4 (8). In the tree from each dataset, a maximum likelihood (ML) analysis was conducted in RAxML 8.2 (38) using the GTRGAMMAI model. Bootstrap values were obtained from 10,000 replicates for the SSU rRNA gene. A Bayesian analysis was performed in MrBayes 3.2.1 (34) using the GTR+I+Γ model. The starting tree was random, and four simultaneous Markov chains in duplicate were run for 10,000,000 generations. Log likelihoods stabilized well before 2,500,000 generations, and the remaining generations were used to measure Bayesian posterior probabilities.

The alignments of bacterial 16S rRNA and *gyrB* gene sequences were concatenated manually, and the ML tree was estimated in RAxML using the GTRGAMMAI model. Parameters and branch lengths were individually optimized for each partition, and bootstrap values were obtained from 10,000 replicates. The GTR+I+Γ model was employed in the Bayesian analysis, and parameters and branch lengths were individually optimized for each partition. The starting tree was random, and four simultaneous Markov chains were run in duplicate for 10,000,000 generations. As described above, log likelihoods stabilized well before 2,500,000 generations, and the remaining generations were used to measure Bayesian posterior probabilities.

Three mitochondrial genes (mt16S rRNA, COI, and COII) were used in host termite phylogeny analyses, as described previously (25). The ML analysis was performed in RAxML using the GTRGAMMA model. Parameters and branch lengths were individually optimized for each partition, and bootstrap values were obtained from 10,000 replicates. A Bayesian analysis was performed in MrBayes using the HKY+Γ model, as described above.

### Cophylogenetic analyses

Cophylogenetic analyses were conducted using five host *Reticulitermes* spp. and 12 samples of the protists and their endosymbionts. Tree topologies reconstructed using the ML method for each phylogenetic analysis were used in the analysis of the congruence of host and symbiont phylogenies. The Jane 4 program with 100 generations and a population size of 100 was used (7). The analysis also tested the significance of topological congruence between the hosts and symbionts. Cophylogeny mapping in Jane 4 employed heuristics to find solutions that minimized the overall cost of a historical reconstruction. The default event costs were as follows: 0 for a codivergence event, 1 for duplication and host switch events, and 2 for loss events. TreeMap (Charleston, M. TREEMAP 3.0 beta. Available at http://sites.google.com/site/cophylogeny) was used to construct the tanglegram for hosts and symbionts.

### Fluorescence *in situ* hybridization (FISH)

FISH was performed to detect endosymbionts, as described previously (21, 24, 35). Briefly, termite gut contents were fixed in 4% paraformaldehyde; fixed cells were then spotted onto a silane-coated glass slide (Matsunami Glass, Osaka, Japan). After dehydration in ethanol, the slides were incubated with the hybridization solution (0.9 M NaCl and 0.1 M Tris-HCl) containing fluorescently-labeled probes at 48°C for 2 h. Specimens were washed for 20 min in a washing buffer (0.2 M NaCl and 0.1 M Tris-HCl) at 48°C. They were then mounted using a Fluoro-Keeper antifade reagent (Nacalai Tesque, Kyoto, Japan) and observed under an Olympus epifluorescence microscope; BX-63 (Olympus, Tokyo, Japan). Probes for the intracellular *Treponema* species and general eubacterial probes (EUB338) were described previously (32). The 5′-termini of the probes were labeled with 6-carboxyfluorescein (6-FAM) or Texas red.

## Results

### Host termites

**Fig. 1:**
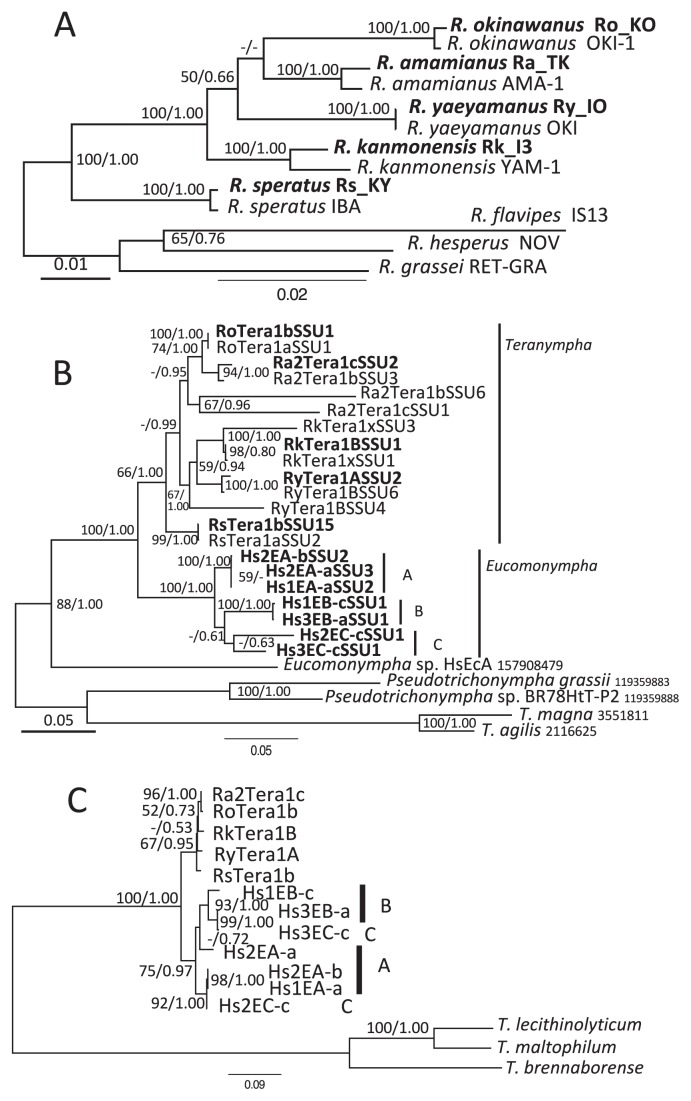
Phylogenetic trees of *Reticulitermes* termites harboring *Teranympha* protists in the hindgut (A), *Teranympha* and *Eucomonympha* protists (B), and endosymbiotic *Treponema* bacteria of *Teranympha* and *Eucomonympha* protists (C). The trees were inferred using RAxML from the concatenated dataset of mitochondrial genes comprising 687, 723, and 658 nucleotide sites of SSU rRNA, cytochrome oxidase (CO) I, and COII, respectively, in (A); from 1515 nucleotide sites of the nuclear SSU rRNA gene, in (B); and from the concatenated dataset of 1447 and 1228 nucleotide sites of the 16S rRNA and *gyrB* genes, respectively, in (C). Separate models with the parameters and branch lengths individually optimized for each gene partition were used for the inference analysis in (A) and (C). Numbers at nodes indicate maximum likelihood bootstrap support as percentages and Bayesian posterior probability values, respectively. Values less than 50% or 0.5 are indicated with hyphens. The outgroup taxa in the analyses were: for (A), three *Reticulitermes* termites (*R. flavipes*, *R. hesperus*, and *R. grassei*) that do not harbor *Teranympha* protists; for (B), two *Pseudotrichonympha* (*P. grassii* and *Pseudotrichonympha* sp.) and two *Trichonympha* protists (*T. agilis* and *T. magna*); and for (C), three *Treponema* species (*T. brennaborense*, *T. maltophilum*, and *T. lecithinolyticum*). The scale bars correspond to 0.01, 0.05, and 0.10 substitutions per site for (A), (B), and (C), respectively. Accession numbers for the gene sequences are shown in supplementary Tables S1 and S2, and Fig. S2 and S3.

Five *Reticulitermes* termites that harbored *Teranympha* protists in their guts were analyzed (Table 1). The distribution of these termites and sampling points are shown in Fig. S1. *Reticulitermes* termites have distinct geographical distributions in Japan, and *H. sjostedti* distributes sympatrically with *R. speratus* and *R. amamianus*. The phylogenetic relationship between these host termites was inferred from the concatenated data of the three mitochondrial genes of 16S rRNA, and cytochrome oxidase subunits I and II. In the ML tree (Fig. 1A), each sequence of the five termite species was closely related to that of the corresponding species in the database, and every species formed a robust monophyletic group. Although the order of their divergence was not fully resolved because of poor bootstrap support in some nodes, the tree topology was congruent with that reported previously (9, 28).

### *Teranympha* and *Eucomonympha* protists

The molecular phylogeny of the gut symbiotic protists of the genera *Teranympha* and *Eucomonympha* was inferred based on their SSU rRNA gene sequences (Fig. 1B). Each *Reticulitermes* termite harbored a morphologically unique *Teranympha* species, and most of the sequences obtained from two independent single protist cells from each termite host typically shared high sequence similarity (>98.0%). However, two sequences sharing 88.0% sequence similarity in *R. amamianus* (Ra2Tera1bSSU3 and Ra2Tera1bSSU6) were obtained from one single-cell sample of *Teranympha*; two sequences (Ra2Tera1cSSU2 and Ra2Tera1cSSU1) sharing 90.0% similarity were obtained from the other sample. Among these four, Ra2Tera1bSSU3 and Ra2Tera1bSSU2 shared more than 98% sequence similarity. We examined the transcribed rRNA sequences by cloning the RT-PCR products from a pool of *Teranympha* cells in this termite; the sequences of fifteen clones were almost identical to one another, and to Ra2Tera1bSSU3 and Ra2Tera1cSSU2 (>98%). *Teranympha* sp. in *R. amamianus* appear to have harbored at least two copies of the SSU rRNA gene in the genome, only one of which was functional. A highly similar sequence was obtained from the two single-cell samples of protists in *R. kanmonensis* and *R. yaeyamanus* (two samples per termite); however, the distantly related sequences, RkTera1xSSU3 and RyTera1BSSU4, respectively, were also obtained and appeared to be the same as that in *R. amamianus*. Consequently, we analyzed the phylogenetic relationships of the protists with sequences of the presumed functional genes only, and the tree topology obtained was used for subsequent comparisons (Fig. S4 and Table S2). Based on these data, *Teranympha* species in *R. okinawanus* and *R. amamianus* were grouped together, and this group was a sister to the group of *Teranympha* species in *R. kanmonensis* and *R. yaeyamanus*. *T. mirabilis* from *R. speratus* formed a clade together with the other *Teranympha* species, but branched out most basally.

In the case of the *Eucomonympha* protists, the sequences from seven single-cell samples were clustered into three sequence groups (A, B, and C in Fig. 1B), although group C was weakly supported. The clones in each group showed high sequence similarities (more than 99%), except for those in group C (94.3%). These sequences were detected in our analyses of hundreds of clones of the SSU rRNA gene amplified by RT-PCR from the whole gut community of *H. sjostedti* as abundant clones (unpublished data), suggesting that the three sequence groups identified herein are functional and represent major populations of *Eucomonympha* spp. in the gut of *H. sjostedti*. These sequences of *Eucomonympha* protists formed a sister group to *Teranympha* species from the five *Reticulitermes* termites. The currently described *Eucomonympha* lineages are paraphyletic (30). As reported previously (4, 30), *Eucomonympha imla* in the wood-feeding cockroach *Cryptocercus punctulatus* is distantly related to *Eucomonympha* spp. in *H. sjostedti*, and the genus of the latter species needs to be reclassified.

### Endosymbiotic bacteria

The 16S rRNA gene sequences of the endosymbiotic bacteria were obtained from each single-cell sample of the *Teranympha* and *Eucomonympha* species. Only a single phylotype affiliated with the genus *Treponema* was detected in each sample. Most of the sequences shared more than 99.5% identity, except for one sample from which a sequence sharing 98.5% identity with others (RkTera1B) was obtained. The representative sequences derived from the same termite species shared high sequence similarities (99.6–100%), while the sequences from other termite species shared lower sequence identities than those from conspecies. As previously reported (32), sequences of the endosymbionts from both protist genera formed a phylogenetic cluster distinct from the ectosymbiont lineages of termite-gut protists in the termite *Treponema* cluster II (14, 32). The endosymbionts of *Teranympha* spp. formed a monophyletic clade, which was clearly separated from the clade formed by the endosymbionts of *Eucomonympha* spp. (Fig. S2).

We also obtained sequences of the *gyrB* gene of the endosymbionts. Since the 16S rRNA gene sequences derived from two single-cell samples of *Teranympha* spp. shared high similarity, only one single-cell sample for each host *Teranympha* termite species was analyzed. Although many of the sequences obtained were closely related to the genomic sequence of the *Treponema* endosymbiont of *Eucomonympha* sp., several distantly related sequences were also obtained; some of these were affiliated with the genus *Treponema* and others were related to those encoded by bacteria from the family Opitutaceae (phylum Verrucomicrobia) or the genus *Ureaplasma* (phylum Tenericutes), both of which are the intracellular bacteria of Eukaryotes. However, except for the *Treponema*-like sequences, frame-shift insertions/deletions or stop codons were detected in these sequences. Since 16S rRNA pseudogenes have been reported in the intranuclear Verrucomicrobia symbionts of their host protist’s genome, and the related intranuclear bacteria are widely distributed in termite-gut protists (36), these *gyrB* pseudogenes appear to be derived from intranuclear symbionts. As in the case of phylogenetic analyses based on the 16S rRNA gene, the *Treponema* endosymbionts formed a monophyletic cluster, and two clades comprising *Teranympha* and *Eucomonympha* endosymbionts were detected (Fig. S2).

In both phylogenetic trees, the branching orders within each clade of the *Teranympha* and *Eucomonympha* endosymbionts were poorly resolved because of their high sequence similarities (Table 2). In the case of the endosymbionts of *Eucomonympha* spp., their phylogenetic relationships did not appear to correlate with the host protist’s phylogeny; the endosymbiont sequences from the same *Eucomonympha* species were not always grouped together. In order to overcome the poor phylogenetic resolution of the single marker gene analysis, the concatenated sequences of 16S rRNA and *gyrB* genes were used in the phylogenetic analysis (Fig. 1C). Although the supporting values at each node were not significant, the *Teranympha* endosymbionts formed a monophyletic clade that was a sister of, but clearly separated from the *Eucomonympha* endosymbionts.

### Topology-based analyses of host-symbiont relationships

**Fig. 2:**
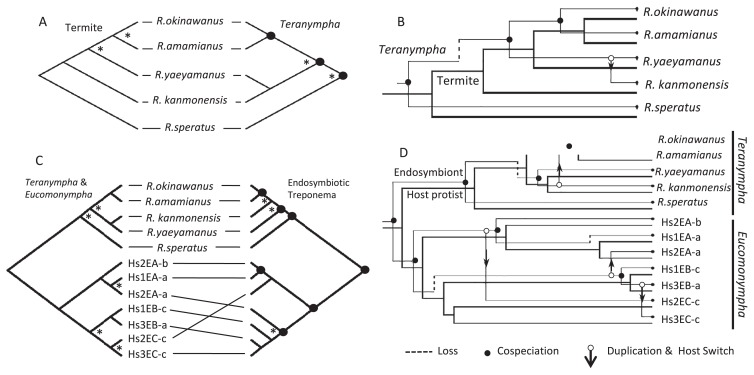
Comparisons of tree topologies between hosts and symbionts. Tanglegrams show cophylogenetic relationships between host termites (left) and *Teranympha* protists (right) in (A), and between protists (left) and their endosymbiotic *Treponema* (right) in (C). The nodes marked with asterisks in (A) and (C) have no significant statistical support (less than 75% Bootstrap or 0.95 BPP). Closed circles in the symbiont trees designate cospeciation events estimated by Jane 4. Coevolutionary reconstructions with the least event costs between the hosts and symbionts are shown in (B) for the host termites and *Teranympha* protists, and in (D) for the host protists and their *Treponema* endosymbionts; thin lines in (B) and (D) correspond to the estimated evolutionary schemes of the symbionts, respectively, with the evolutionary events of cospeciation, loss, and duplication and the host switch indicated.

The host and symbiont phylogenies reconstructed by the ML method were used to assess their phylogenetic congruence (Fig. 2). The tanglegram and relationships among the host *Reticulitermes* termites and *Teranympha* protists revealed a significant coevolutionary congruence, although the tree topologies did not perfectly match (Fig. 2A). The evolutionary event that was incongruent in the two trees occurred at a weakly supported node. We then used Jane 4 (7) to generate coevolutionary events with the lowest cost sets (Fig. 2B). A significant global cost (*P*=0.02) was observed, which was 3 for cospeciation; 0 for duplication; 1 for host-switch; 1 for loss; and 0 for failure to diverge between the host termites and *Teranympha* protists (Table 3).

Reconciling the phylogenies of host protists and their endosymbionts indicated a significant topological congruence (*P*=0.04), with seven codivergence events out of a possible 11, four host switches, and three losses (Table 3). Similar to the relationship between the host termites and their gut protists, the tree topologies of the *Teranympha* protists and their endosymbionts were largely congruent with three codivergence events out of a possible four (Fig. 2C and D), and incongruence was observed at a weakly supported node. In contrast, the coevolutionary relationship between the *Eucomonympha* protists and their endosymbionts was complex, with three host switch events (Fig. 2C and D). These incongruence events occurred at strongly supported nodes of the endosymbiont tree. The endosymbiont of the host protist group C (Hs2EC-c) was closely related to the endosymbionts of the two host protists in group A (Hs1EA-a and Hs2EA-b), and the endosymbionts of the host protists in groups B and C (Hs3EB-a and Hs3EC-c, respectively) were also closely related (see also Fig. 1C).

### *In situ* identification of *Teranympha* endosymbionts and their morphology

**Fig. 3:**
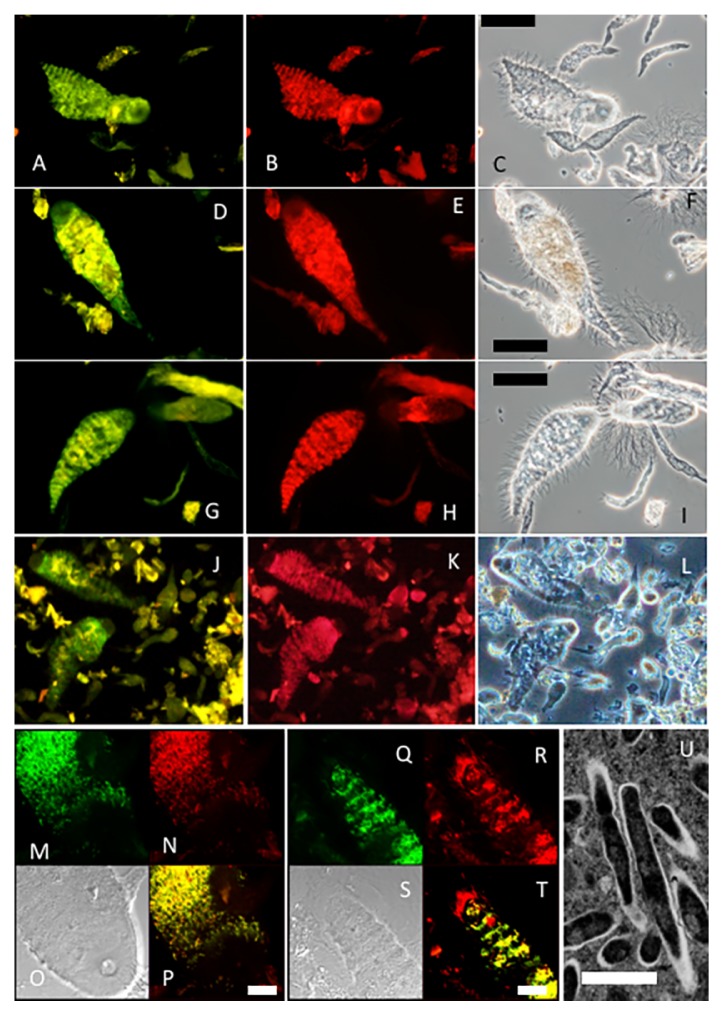
*In situ* detection of the endosymbiotic *Treponema* of *Teranympha* spp. in *R. amamianus* (A–C), *R. kanmonensis* (D–F), *R. okinawanus* (G–I), *R. yaeyamanus* (J–L), and *R. speratus* (M–U). The specific probe IIC-637 to the 16S rRNA gene sequences of endosymbiotic *Treponema* labeled with 6-FAM (A, D, G, J, M, and Q) was used simultaneously with the eubacterial consensus probe labeled with Texas red (B, E, H, K, N, and R). Panels C, F, I, and L show phase-contrast micrographs of hindgut content samples. Images in panels M–T were acquired using confocal laser scanning microscopy, with the visualized fluorescent signals. Panels O and S show differential interference contrast micrographs. A merged image of panels M and N is shown in panel P; that of panels Q and R is shown in panel T. A transmission electron micrograph of the endosymbionts in a *T. mirabilis* cell is shown in panel U. The amorphous yellow signals in panels A, D, G, and J are autofluorescence signals, mostly originating from wood particles incorporated into the protist cells by phagocytosis. Bars correspond to 50 μm in panels C, F, I, and L; 10 μm in panels P and T; and 1 μm in panel U.

A previously used oligonucleotide probe (IIC-637) targeting the 16S rRNA of the *Treponema* endosymbionts of *Eucomonympha* spp. was used to detect the endosymbionts of *Teranympha* spp. by FISH because, as shown above, the targeted sequence was conserved (32). This probe annealed to the rod-shaped endosymbiotic bacteria of *Teranympha* protists in the gut of all *Reticulitermes* termites examined (Fig. 3A, D, G, J, M, and Q). The same rod-shaped endosymbionts were detected during simultaneous hybridization of a universal bacterium probe (Fig. 3B, E, H, K, N, and R). Although the universal probe also detected ectosymbiotic bacteria, *e.g.*, spirochetes attached at the posterior end of *Teranympha* cells (Fig. 3R), both probes simultaneously detected all bacteria in the protist cytoplasm. Most of the *Teranympha* cells harbored endosymbiotic bacteria that were homogeneously and densely packed in the cytoplasm, except for the anterior end of the cell, the rostrum (Fig. 3A, D, G, J, M, and Q). The endosymbionts of *T. mirabilis* in the gut of *R. speratus* (Fig. 3M, N, O, P, Q, R, S, T, and U) appeared to be more densely packed than *Teranympha* spp. in other termites. The *Teranympha* protists in *R. yaeyamanus* appeared to be colonized to a lesser extent, with the endosymbionts being restricted to the anterior of the cells, except for the rostrum (Fig. 3J, K, and L).

Transmission electron microscopy of ultrathin sections of *T. mirabilis* revealed the presence of numerous endosymbiont cells in the cytoplasm of *T. mirabilis* (Fig. 3U), as previously reported (32). The endosymbionts were 0.42±0.07 μm in width (mean±standard deviation) and 2.09±0.29 μm in length (*n*=9), and were longer than the *Eucomonympha* endosymbionts (1.33±0.31 μm×0.41±0.05 μm) (32). Endosymbiont cells possessed a single cell membrane and were surrounded by an external membrane that may have been derived from the host protist, as in the case of the endosymbionts of *Eucomonympha* sp. (Fig. 3U).

## Discussion

Cophylogenetic analyses of gut protists from the family Teranymphidae, and their endosymbionts in five *Reticulitermes* termites and *H. sjostedti* revealed their possible cospeciating evolutionary relationships, particularly between the triplex symbiotic partners of *Teranympha* protists, their endosymbionts, and their host termites. Incongruence in evidence against a completely cospeciating relationship was observed, but occurred at nodes showing poor resolution, *i.e.*, for the relationship between *R. yaeyamanus* and *R. kanmonensis*, or between their symbionts. Therefore, we consider cospeciation to be the most plausible evolutionary scenario, albeit with the possibility of deviations.

A similar cospeciating relationship between the triplex symbiotic partners was reported for *Pseudotrichonympha* protists, their Bacteroidales endosymbionts, and their host termites from the family Rhinotermitidae (except for the genus *Reticulitermes*) (25). As previously discussed, the strict vertical transmission of gut symbionts is likely warranted by the proctodeal feeding behavior that characterizes the sociality of termites (31).

The termite gut fauna is discarded at every molting, but is acquired from nestmates; it is also carried over to a newly founded colony by the primary termite reproductives (31). Since endosymbionts are abundant in the protist cell, the cell is stably transmitted during protist cell division. The crucial roles played by the protists and their endosymbionts in their host termites, *i.e.*, cellulose decomposition by the former and nutrient supply by the latter, appear to have had a significant impact on the maintenance of these symbiotic partners and their mutual adaptation.

In contrast to *Pseudotrichonympha*, which are widely distributed among termites from the family Rhinotermitidae, the *Teranympha* protists have only been found in *Reticulitermes* termites and are exclusively limited to the Asian species in this genus; however, the composition of other protist species in all *Reticulitermes* termites appears to be similar (17). In the present study, we confirmed this close phylogenetic relationship, which suggests a possible common ancestor of *Teranympha* spp. and *Eucomonympha* spp. in the *H. sjostedti* termite. This observation also supports the previously proposed horizontal transfer of the gut fauna between *Reticulitermes* and *Hodotermopsis* termites (18). If this is the case, the absence of *Teranympha* likely represents a secondary loss after fauna transfer, with other members of the gut fauna possibly complementing the symbiotic roles of *Teranympha* and its endosymbionts. Furthermore, the *Treponema* endosymbionts of *Teranympha* spp. and *Eucomonympha* spp. form a closely related sister group. The results of our analyses imply that an ancestor of both protist groups acquired the endosymbiont before fauna transfer and that, after this transfer, the protists and their endosymbionts were vertically inherited together over a long evolutionary time period and cospeciated according to the species differentiation of the host termites.

In *Teranympha* and *Pseudotrichonympha* cases, only a single protist species appeared to be present in the gut microbial community of a single host termite species. This may affect the cospeciating evolutionary process because the spatial separation of host protist lineages creates the potential for accepting endosymbionts; hence, the opportunity for a host switch does not exist. In contrast, *Eucomonympha* spp. are sympatric and present simultaneously within the gut community of a single host termite. Although cospeciation appears to be a general rule for the *Teranympha* and *Eucomonympha* protists and their endosymbionts, as shown by the overall significance in the cophylogeny analysis (Table 3), the phylogenies of *Eucomonympha* spp. and their endosymbionts are not always congruent. For example, the endosymbiont phylotype Hs2EC-c from group-C protists was more closely related to those from group-A protists than to Hs3EC-c. Furthermore, the endosymbiont phylotypes Hs3EB-a and Hs3EC-c from protist groups-B and -C, respectively, were very closely related, and the endosymbiont phylotype Hs2EA-a from group-A protists was distantly related to the other endosymbionts from group-A protists. These examples of incongruence in evidence against cospeciation suggest more frequent host switches of endosymbionts in *Eucomonympha* spp. than in *Teranympha* spp., possibly because multiple protist species are present in the same gut community. A similar host switch-like relationship was reported for *Trichonympha* sp. and its endosymbiont “*Ca.* Endomicrobium trichonymphae” in the gut of *H. sjostedti*, with multiple *Trichonympha* species being present in the gut of this termite (15). However, this host switch-like relationship is the only exception among the strict cospeciating relationships of *Trichonympha* protists and *Endomicrobium* endosymbionts. The genomes of the endosymbionts of *Trichonympha* and *Pseudotrichonympha* are small in size, likely as a consequence of reductive evolution (12, 13). These endosymbiont species with reduced genomes may be more dependent on the host protists than the endosymbionts of *Eucomonympha*, and, thus, their opportunity for a host switch may be reduced. In contrast, the genome size of the endosymbiont of *Eucomonympha* was not as severely reduced, although its gene content, as evaluated by a cluster of orthologous group analysis, was similar to the genomes of the *Trichonympha* and *Pseudotrichonympha* endosymbionts; for example, the relative abundance of cell motility genes is universally lower, and that of the translation and coenzyme metabolism genes is universally higher in the genomes of the endosymbionts of termite-gut protists than in cultured treponemes (32). The acquisition of the endosymbiont by *Eucomonympha* protists appears to be a recent evolutionary event, and the endosymbiont species is at an initial developmental stage of adaptive evolution. The endosymbiont of *Eucomonympha* may be more independent of the host protists than the other two, and may be able to switch the host lineages more frequently.

In the present study, we confirmed that *Treponema* endosymbionts are widely and commonly distributed among the *Teranympha* and *Eucomonympha* protists examined to date. As discussed above and previously (32), *Treponema* endosymbionts are monophyletic and appear to have originated from an ectosymbiont lineage of termite-gut protists. Nevertheless, the phylogenetic analyses revealed that the endosymbionts of *Teranympha* spp. clearly form a distinct clade from those of *Eucomonympha* spp. The sequence similarity of the 16S rRNA gene of the endosymbionts of *Teranympha* species (intra-genus) was greater than 99.25%, whereas the sequence similarity of the endosymbionts between *Eucomonympha* and *Teranympha* species (inter-genera) was less than 97.1% (Table 2). It is generally accepted that bacteria sharing less than 98.7% 16S rRNA gene sequence similarity need to be classified into distinct species because this sequence similarity corresponds to a DNA reassociation value less than 70%, *i.e.*, a threshold of bacterial species delineation (37). The shared sequence similarity of the *gyeB* gene of the endosymbionts was also high among *Teranympha* spp., but low between *Teranympha* and *Eucomonympha* protists (Table 2). Based on these findings, we propose classifying the endosymbiont species of *Teranympha* into a species distinct from ‘*Ca.* T. intracellularis’, the endosymbiont of *Eucomonympha* spp. (32). Therefore, we propose a novel species “*Candidatus* Treponema teratonymphae” for *Teranympha* endosymbionts. Although we used the popular original name of the genus “*Teranympha*” in the present study, its erroneous orthography was pointed out earlier (*Teratonympha* is the proper name) (11), and we adopted the proper form for the nomenclature of the *Treponema* endosymbiont species.

### Description of “*Candidatus* Treponema *teratonymphae*”

Te.ra.to.nym’phae. N.L. gen. n. teratonymphae, of Teratonympha, referring to the generic name of the host protist. Cells are rods, 1.64–2.39 μm×0.32–0.51 μm in size (average 2.09±0.29 μm×0.41±0.07 μm). They lack flagella and are surrounded by two membranes; the outer membrane is presumably the host-derived membrane. The bacterium is specifically found in the cytoplasm of the parabasalian protist *Teranympha* (or its amended *Teratonympha*) spp., in the hindgut of *Reticulitermes* spp. termites. Thus far uncultured, but forms a monophyletic group based on sequence analyses of the 16S rRNA and *gyrB* genes (DDBJ accession numbers: LC276704–LC276733).

## Supplementary Material



## Figures and Tables

**Table 1:** Host termite species and single-cell samples of protist species used in gene identification and phylogenetic analyses.

Termite	Sampling location	Protist	Sample name
*R. speratus*	Kofu, Yamanashi	*Teranympha mirabilis*	RsTera1a, RsTera1b
*R. amamianus*	Tokunoshima, Kagoshima	*Teranympha* sp.	Ra2Tera1b, Ra2Tera1c
*R. kanmonensis*	Shimonoseki, Yamaguchi	*Teranympha* sp.	RkTera1B, RkTera1x
*R. yaeyamanus*	Iriomote, Okinawa	*Teranympha* sp.	RyTera1A, RyTera1B
*R. okinawanus*	Kunigami, Okinawa	*Teranympha* sp.	RoTera1a, RoTera1b
*H. sjostedti*	Yakushima, Kagoshima	*Eucomonympha* sp.	A Hs1EA-a, Hs2EA-a, Hs2EA-b
		*Eucomonympha* sp. B	Hs1EB-c, Hs3EB-a
		*Eucomonympha* sp. C	Hs2EC-c, Hs3EC-c

**Table 2:** Nucleotide sequence similarities of 16S rRNA and *gyrB* genes of the endosymbiotic *Treponema* of the intra-genus or inter-genus of Teranymphidae protists

	16S rRNA (%)	*gyrB* (%)
*Teranympha* endosymbiont	99.25–99.73	97.23–99.35
*Eucomonympha* endosymbiont	98.64–99.86	92.02–99.92
*Teranympha* and *Eucomonympha* endosymbiont	96.46–97.07	90.23–91.86

**Table 3:** Number of event types required for the reconciliation of host and symbiont trees

Host and symbiont	Total cost	Cospeciation	Duplication	Host switch	Losses/sorting	Failure to diverge	*P*-value
Termite and *Teranympha*	3	3	0	1	1	0	0.02*
Protist and endosymbiont	11	7	0	4	3	0	0.04*

*P*-values were computed from 999 random reconstructions. Asterisks indicate a 5% level of significance. The event costs used for the analyses were as follows: 0 for cospeciation; 1 for duplication; 2 for host switching; 1 for sorting; and 1 for failure to diverge.
