# Supplementary material for: Host-Symbiont Cospeciation of Termite-Gut Cellulolytic Protists of the Genera Teranympha and Eucomonympha and their Treponema Endosymbionts
Source: Microbes Environ. 2018 Mar 29;33(1):26–33. doi: 10.1264/jsme2.ME17096 (PMC5877339; doi:10.1264/jsme2.ME17096)
Supplement: Supplementary file 1 [file 33_26_s1.pdf]

Table S1. List of termite taxa used in this study and accession numbers of the genes.

Termite	Sample name	Genes: mt 16S rRNA	mt COI	mt COII	Reference
R. amamianus	AMA-1	KM245682	KM245745	KM245808	10
	Ra_KT	LC276741	LC276746	LC276751	this study
R. kanmonensis	YAM-1	KM245685	KM245748	KM245811	10
	Rk_I3	LC276742	LC276747	LC276752	this study
R. okinawanus	OKI-1	KM245689	KM245752	KM245815	10
	Ro_OK	LC276743	LC276748	LC276753	this study
R. speratus	IBA	KM245697	KM245760	KM245823	10
	Rs_YK	LC276744	LC276749	LC276754	this study
R. yaeyamanus	OKI	KM245699	KM245762	KM245825	10
	Ry_OI	LC276745	LC276750	LC276755	this study
R. flavipes	IS13	(NC_009498)			Genome. 50 (2), 188-202 (2007)
R. grassei	RET-GRA	(KU925237)			Proc.Biol.Sci.283(1827) 2016
R.hesperus	NOV	KM245644	KM245707	KM245770	10

Table S2 *Teranympha* and *Eucomonympha* species used for gene identification and phylogenetic analyses.

Termite	Protist	Sample	Protist SSU rRNA	Endocymbionts 16S rRNA	Endocymbionts *gyrB*
R. amamianus	*Teranympha* sp.	Ra2Tera1b	LC276683, LC276684	LC276704	
		Ra2Tera1c	LC276685, LC276686*	LC276705*	LC276721*
R. kanmonensis	*Teranympha* sp.	RkTera1B	LC276687*	LC276706*	LC276722*
		RkTera1x	LC276688, LC276689	LC276707	
R. okinawanus	*Teranympha* sp.	RoTera1a	LC276690	LC276708	
		RoTera1b	LC276691*	LC276709*	LC276723*
R. speratus	*Teranympha milabilis*	RsTera1a	LC276692	LC276710	
		RsTera1b	LC276693*	LC276711*	LC276724*
R. yaeyamanus	*Teranympha* sp.	RyTera1A	LC276694*	LC276712*	LC276725*
		RyTera1B	LC276695, LC276696	LC276713	LC276726
H. sjostedti	*Eucomonympha* sp. A	Hs1EA-a	LC276697*	LC276714*	LC276727*
		Hs2EA-a	LC276699*	LC276716*	LC276729*
		Hs2EA-b	LC276700*	LC276715*	LC276730*
	Eucomonympha sp. B	Hs1EB-c	LC276698*	LC276717*	LC276728*
		Hs3EB-a	LC276702*	LC276719*	LC276732*
	Eucomonympha sp. C	Hs2EC-c	LC276701*	LC276718*	LC276731*
		Hs3EC-c	LC276703*	LC276720*	LC276733*

Asterisks indicate the sequences used for the cophylogenetic analyses and the sequence concatenation of 16S rRNA and *gyrB* genes.

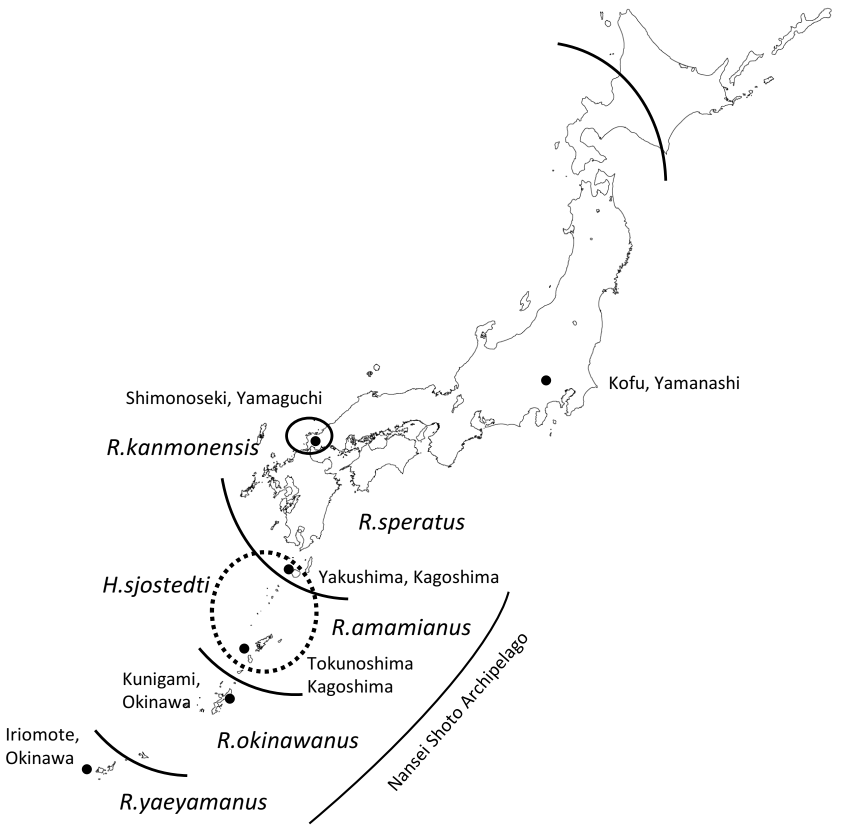

Figure S1 . Distribution of *Reticulitermes* spp. and *Hodotermopsis* termites in Japan. Solid circles indicate sampling points in the current study. Areas encircled with dotted and solid lines indicate the habitat of *H. sjostedti* and *R. kanmonensis*, respectively. The semicircles indicate the northern and southern habitat boundaries of each termite species.

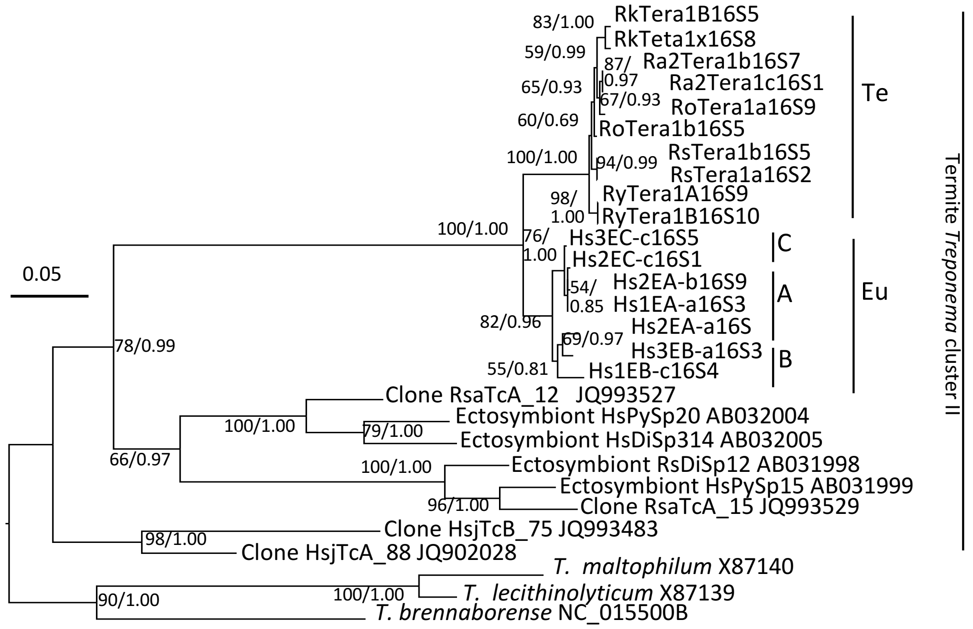

Figure S2. Phylogenetic relationships of endosymbiotic *Treponema* based on the 16S rRNA gene sequence. The tree was inferred in RAxML, based on 1447 nucleotide sites. Clone sequences identified as ectosymbionts of the termite-gut protists and obtained from isolated cells of termite-gut protists are included in the phylogenetic inference. Three *Treponema* species, *T. brennaborense*, *T. maltophilum*, and *T. lecithinolyticum* were used as outgroups. Accession numbers of these reference sequences are indicated in the tree. The supporting values (bootstrap in RAxML/Bayesian posterior probability) are indicated at the nodes. Values below 50% or 0.5 are indicated with hyphens. The scale bar corresponds to 0.05 substitutions per site.

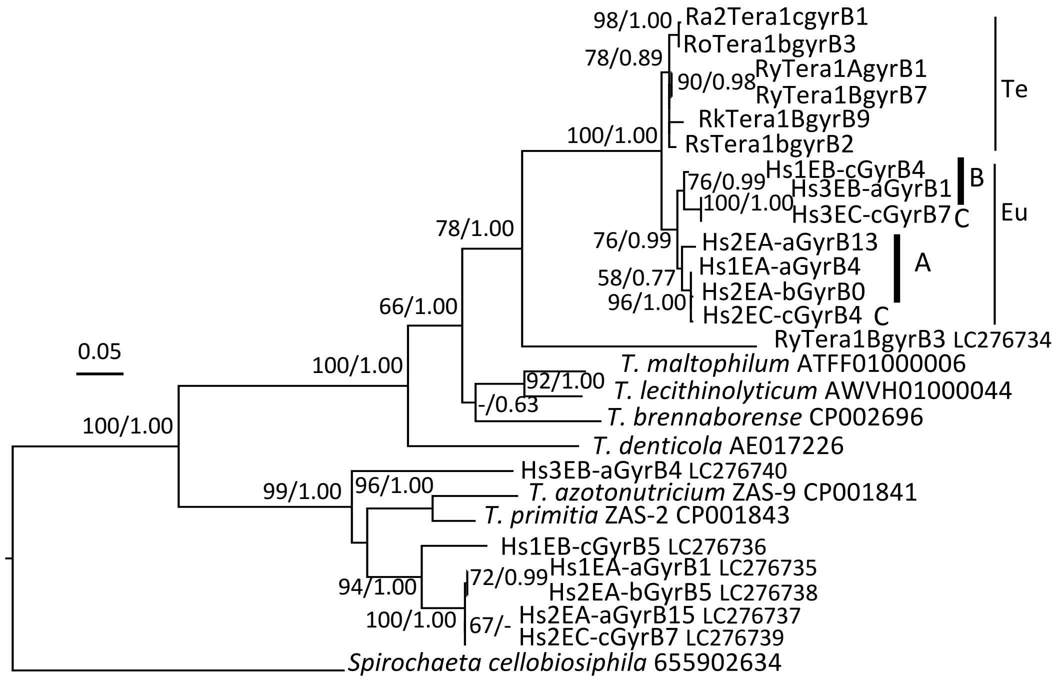

Figure S3. Phylogenetic relationships of endosymbiotic *Treponema* based on the predicted GyrB amino acid sequences. The tree was inferred using a LG+I+G model in RAxML, based on 392 amino acid sites. Gene sequences of several *Treponema* species and sequences obtained from single-cell samples of *Eucomonympha* spp. that were distantly related to the endosymbionts of *Teranympha* and *Eucomonympha* but belonged to *Treponema* were included in the phylogenetic inference; their accession numbers are indicated. *Spirochaeta cellobiosiphila* was used as an outgroup. The supporting values (bootstrap in RAxML/Bayesian posterior probability) are indicated at the nodes. Values below 50% or 0.5 are indicated with hyphens. The scale bar corresponds to 0.05 substitutions per site.

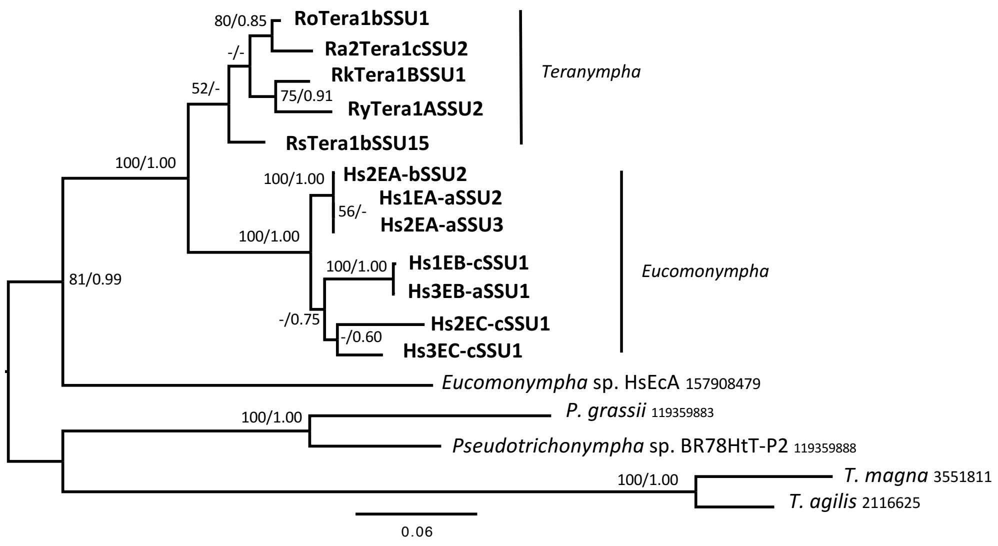

Figure S4. Phylogenetic tree of *Teranympha* and *Eucomonympha* protists. The tree was inferred using RAxML from 1515 nucleotide sites of the nuclear SSU rRNA gene. Numbers at nodes indicate maximum likelihood bootstrap support as percentages and Bayesian posterior probability values, respectively. Values below 50% or 0.5 are indicated with hyphens. The outgroup taxa in the analyses were two *Pseudotrichonympha* (*P. grassii* and *Pseudotrichonympha* sp.) and two *Trichonympha* protists (*T. agilis* and *T. magna*). The scale bars correspond to 0.06 substitutions per site.
